# Prevalence of Traditional Asian Postpartum Practices at a Federally Qualified Health Center

**DOI:** 10.1007/s10903-021-01299-0

**Published:** 2021-12-14

**Authors:** Van Viet Thuy Nguyen, Micha Y. Zheng, Stephanie M. Liu, Michael A. Kallen, Kerry Kay, Susan L. Ivey

**Affiliations:** 1 Present Address: San Jose, USA; 2 Present Address: San Francisco, USA; 3 The Permanente Medical Group, Inc., 1800 Harrison Street, Oakland, CA 94609 USA; 4 Department of Medical Social Sciences, Feinberg School of Medicine, Northwestern University, Chicago, IL 60611 USA; 5 Asian Health Services, Oakland, CA 94607 USA; 6 UC Berkeley-UCSF Joint Medical Program, School of Public Health, University of California, 570 University Hall #1190, Berkeley, CA 94720 USA; 7 Present Address: Missouri City, USA; 8 Present Address: Oakland, USA

**Keywords:** Doing the month, Sitting the month, Traditional Chinese Medicine, Postpartum ritual

## Abstract

To evaluate the knowledge of, participation in, attitudes towards, and experiences with “doing the month” (DTM), a traditional Chinese and Vietnamese postpartum practice, at a federally qualified health center that serves predominantly Asian immigrants. DTM practices revolve around the balance between yin and yang and include practices such as the mother remaining on bed rest for as long as possible, restricting diet to certain foods, and avoiding visitors and social activities. A cross-sectional survey in Chinese, Vietnamese, and English was developed to determine the prevalence of women who have heard of and participated in DTM. 154 respondents participated. The mean age of respondents was 40.1 years. Without prompting of what DTM was, 58 (37.7%) responded that they had heard of DTM. After an explanatory paragraph, this increased to 117 (76.6%) participants. Out of 107 patients who have children, 65 (60.7%) “did the month” after giving birth. Participation rates were highest for women who identified as Chinese or Vietnamese. Likert-type scale questions showed that respondents believed DTM was stressful but enjoyable and helpful for recovery from childbirth. In conclusion, DTM is a common practice that health providers should be aware of.

## Background

Chinese and Vietnamese immigrants make up a substantial number of immigrants in the United States (U.S.). Chinese immigrants are the third-largest foreign-born group in the U.S., after Mexican and Indian immigrants. Since 1980, the population of Chinese immigrants has grown more than sixfold, reaching 2.3 million in 2016, or 5% of the nation’s immigrants. The Chinese immigrant population is highly concentrated in California (31%) and New York (20%) [1]. Vietnamese immigrants represent 3% of the nation’s immigrants and are the sixth largest immigrant group after immigrants from Mexico, India, China, the Philippines, and El Salvador. Most Vietnamese immigrants settle in California (39%), Texas (13%), Washington State (4%), and Florida (4%) [2]. As people immigrate, they often bring cultural practices and traditions to their new countries, with one example as “doing the month (DTM).”

“DTM” is rooted in Traditional Chinese Medicine (TCM). TCM describes two opposing forces: yin (hot air, activity, or positive energy) and yang (cold air, passivity, or negative energy). According to TCM, yin and yang forces regulate the entire universe, including the human body, and health is correlated with a proper balance of qi, the life energy. After giving birth, TCM theories state that a woman’s harmony and balance of yin and yang are impaired, leading to a physiologically vulnerable state [3]. Because of this imbalance, Chinese women are often encouraged to participate in a postpartum recuperation period commonly known as “坐月子” (Mandarin: “zuo yue zi,” Cantonese: “cho yet,” or Taiwanese: “tso-yueh-tzu”), directly translated as ‘‘DTM’’; that is, they “do the month” from one month to forty days after childbirth in order to heal from their childbearing experience [4]. During this healing time, a woman is supposed to be on bed rest for as long as possible; her mother or mother-in-law takes over childcare, cooking, and domestic work. The woman must avoid certain activities, including taking a bath or shower, participating in social activities, seeing visitors, going out of the house, having sexual intercourse, being exposed to water that has not been boiled, being exposed to windy drafts, brushing her teeth, and washing her hair. The woman must also follow a strict diet that restores her yin and yang equilibrium, such as avoiding cold foods (e.g., cold drinks, ice cream, certain fruits, and vegetables) and eating more hot foods or foods that remove cold (e.g., boiled eggs, chicken, fish soup, ginger, dates, chicken, pig’s feet, internal organs). The mother is also expected to have plenty of rest while someone else takes care of the baby and to keep herself warm, drink only hot liquids, stay away from strangers, and cover her head to prevent chills [5–15].

While DTM has been practiced for hundreds of years, it may conflict with routine postpartum care in the U.S. healthcare system [16]. The American College of Obstetricians and Gynecologists (ACOG) recommends that women have at least two postpartum appointments within 12 weeks of childbirth, with the first of these appointments being scheduled within the first 3 weeks after delivery [17]. Following this recommendation will require women to shorten or temporarily pause DTM to leave her home for the initial appointment. For women with chronic medical conditions like diabetes, hypertensive disorders, and mood disorders, which can be complicated by postpartum changes to the body, DTM may prevent timely care that would otherwise occur during early obstetrician-gynecologist appointments. If the mother is breastfeeding and taking medications, these appointments also provide an opportunity for the obstetrician-gynecologist to review the woman’s medications to ensure they do not contain agents that can enter her breastmilk and are safe for infants [17].

In mainland China, Taiwan, Hong Kong, and Vietnam, “DTM” is still a common practice [15]. Women usually return to their family home after giving birth to receive support from their mothers. DTM practices are also common among the Chinese diaspora [18, 19]. There are, surprisingly, no published studies of the prevalence of participation in this practice in the U.S. The purpose of this study was to determine participant knowledge of “DTM” as a postpartum practice, detect the prevalence of participation in “DTM,” assess the attitudes and beliefs towards this practice, and understand participant experiences with this practice among Asian American women, particularly Chinese and Vietnamese women, encountered at a federally qualified health center in Oakland, California.

## Methods

The population studied were patients at a federally qualified health center that serves predominately Asian immigrants and refugees. A literature review of “DTM” revealed the practice’s origins and various components. The authors used this information to develop a cross-sectional survey for this study. The language-specific versions of the survey were piloted and validated by two women fluent in English and either Chinese or Vietnamese. Final item phrasing in English and ordering were validated and completed by consultation with a psychometrician. There was a maximum of 43 questions that could be answered, depending on participants’ answers and the survey’s subsequent branching or skip logic. The key domains assessed in the full-length survey included: knowledge of, prevalence of, experiences with, and attitudes towards “DTM.” Knowledge of DTM was assessed by first asking participants if they had heard of “DTM” or “sitting the month,” followed by an explanation of what “DTM” is before asking participants again if they had heard of the practice. Prevalence was detected by asking participants about their plans to participate in or history of participating in DTM. Experiences with DTM were assessed by asking participants about stress from, facilitators/barriers to, and provider knowledge about DTM. Lastly, attitudes toward DTM were assessed by asking participants whether they enjoyed “DTM” and whether they found it helpful to recovery after childbirth.

From June to August 2017, female patients in the waiting room of a federally qualified health center in Oakland, California, were asked if they would be willing to complete an iPad-based Qualtrics survey, receiving a $5 grocery store gift card upon its completion. Both recruitment scripts and the survey itself were available in Simplified/Traditional Chinese, Vietnamese, and English. Participant recruiters were fluent in Mandarin Chinese, Vietnamese, and/or English. There were a few female patients able to read and/or speak Mongolian, Hmong, Spanish, and Russian who showed interest in participating but were unable to do so because the survey was not translated into those languages, and no participant recruiters spoke those languages to translate the survey for them. The first page of the survey was an informed consent page, allowing informed consent to be obtained from each study participant prior to allowing the participant to continue her survey. This study was approved by the Institutional Review Board.

Survey data were collected and downloaded from Qualtrics. Data from 154 completed surveys were initially cleaned in Microsoft Excel 2016. Additional cleaning and analysis were performed in SAS 9.4, SAS Institute Inc., Cary, NC, U.S. Chi-square and Fisher’s exact tests were performed to test for associations, with statistical significance set at the p-value < 0.05 level. Phi and Cramer’s V statistics were obtained to estimate effect sizes for the 2 × 2 and larger contingency tables, respectively. To interpret Phi and Cramer’s V values, an effect with an absolute value equal to or greater than 0.5 was considered large, an effect with an absolute value equal to or greater than 0.3 and less than 0.5 was considered medium, and an effect with an absolute value equal to or greater than 0.1 and less than 0.3 was considered small. The effect size can be positive or negative, where positive indicates agreement while negative indicates disagreement (e.g., + 1.00 would be perfect agreement, while − 1.00 would be perfect disagreement). Some multi-item scales were created, and they were then tested for internal consistency reliability, using a criterion of Cronbach’s alpha ≥ 0.70 to proceed with analytic use of a scale. Some categories of multi-category demographic variables were collapsed for analysis due to sample size constraints and the need for robust, interpretable results. Analysis of variance (ANOVA) was used to evaluate differences in scale means among demographic variable-based groups, with statistical significance set at the p value < 0.05 level. Post hoc testing (Tukey’s paired comparisons) was conducted when appropriate (i.e., when an ANOVA’s omnibus F test was statistically significant and more than two groups were being compared). Cohen’s D statistic was obtained as the effect size estimate, with values equal to or greater than 0.8 considered large effects, values equal to or greater than 0.5 and less than 0.8 considered medium effects, and values equal to or greater than 0.2 and less than 0.5 considered small effects.

## Results

A total of 154 women participated. The mean age of this population is 40.1 years (SD = 15.5). For those women who had given childbirth, participant demographics and their frequency of participating in DTM are shown in Table 1.

**Table 1:** Respondent demographics and univariate results (agreement with “I Have Participated in Doing the Month Practices” out of all participants who have given childbirth)

Variable	Variable category	Frequency of agreement	Percent of agreement (Effective sample size/Total sample size × 100) (%)	p-value	Effect size (Phi, Cramer’s V)
Age (in years)	17–44	34/54	63.0	.343	NA
	45–59	18/35	51.4		
	60 +	13/18	72.2		
Place of birth	China or Hong Kong	29/33	87.9	.003	0.415^b^
	U.S.	5/8	62.5		
	Vietnam	16/27	59.3		
	Other^b^	5/12	41.7		
	Southeast Asia^a^	10/27	37.0		
Self-identified race/ethnicity	Chinese^c^	35/39	89.7	< .001	0.504^n^
	Vietnamese	17/28	60.7		
	Southeast Asian^d^	9/27	33.3		
	Other^e^	4/13	30.8		
Year immigrated to the U.S.	1975–2000	14/27	51.9	0.552	NA
	2001–2017	46/72	63.9		
	Non-immigrant	5/8	62.5		
Primary language spoken at home	Chinese^f^	35/40	87.5	< .001	0.469 ^b^
	Vietnamese	15/25	60.0		
	SE Asian Language^g^	6/20	30.0		
	English	5/12	41.7		
	Other^h^	4/10	40.0		
Self-reported spoken English level	Not at all	12/14	85.7	0.073	NA
	Not well	28/45	62.2		
	Well	14/31	45.2		
	Very well	11/17	64.7		
Highest level of school completed	Elementary school or less	16/22	72.7	.267	NA
	Middle school to high school	32/52	61.5		
	Some college	12/20	60.0		
	Bachelor’s degree or more	5/13	38.5		
Marital status	Never married	9/14	64.3	1.000	NA
	Living with partner	4/6	66.7		
	Married	41/68	60.3		
	Separated, divorced, or widowed	11/19	57.9		
Health insurance coverage	Public^i^ or not insured	54/94	57.5	.074	NA
	Private^j^	11/13	84.6		
Total respondents		**107**			

*NA* not applicable

^a^Total N varied per category within variables. For example, “69/96” indicates 69 out of a possible 96 women agreed

^b^Medium effect size

^c^SE Asia place of birth does not include Vietnam; SE Asia place of birth includes Burma, Cambodia, Laos, Malaysia, Philippines, Thailand

^d^Other place of birth includes Afghanistan, Honduras, Korea, Mexico, Mongolia, Uzbekistan

^e^Chinese race/ethnicity includes Chinese, Hong Kongese, Shanghainese, and Taiwanese

^f^Southeast Asian race/ethnicity does not include Vietnamese; SE Asian includes Burmese, Thai, Cambodian, Filipino, Karen, Laotian, Mien

^g^Other race/ethnicity includes Farsi, Russian, Uzbek, Mongolian, Spanish

^h^Small effect size

^i^Chinese language includes Cantonese, Mandarin, and Taishanese

^j^SE Asian language does not include Vietnamese; includes Burmese, Cambodian, Kachin, Karen, Karenni, Khmer, Laotian, Mien, Pangasinan, S’Gaw Karen, Tagalog, Thai

^k^Other language includes Farsi, Mongolian, Russian, Uzbek, Spanish

^l^Public health insurance includes Health Pac, Medi-Cal/Medicaid, Medicare, VA, Alameda Alliance

^m^Private insurance includes purchased directly from an insurance company, through employer or union, student health insurance, and Covered California

^n^Large effect size

Before any prompting, 37.7% of all participants responded “yes” when asked if they had ever heard of the practice, “DTM.” After reading an explanatory paragraph that gave clear examples of the practice, the number of participants who responded “yes” to that question almost doubled, to 76.0%.

### Prevalence

Participants were asked, “Do you know anyone who has done the month (for example: your mother, siblings, etc.)?” Out of 117 respondents who had heard of DTM after the explanatory paragraph, 82 (70.1%) said “yes,” 25 (21.4%) said “no,” 4 (3.4%) said “not sure,” and 6 (5.1%) did not respond.

Participants were also asked, “After giving birth, did you do the month?” Out of 107 participants who had children, 65 (60.7%) said “yes,” 42 (39.3%) said “no.”

### Doing the Month’s Effects on Appointment Adherence

Out of the participants who had “done the month” (n = 65), when asked if they felt that “DTM” prevented them from seeing their doctor the month after giving birth, 10 (15.4%) said “always” or “usually,” 5 (7.7%) said “sometimes,” 44 (67.7%) said “rarely” or “never,” and 6 (9.2%) said “not applicable.”

### Barriers to Doing the Month

Out of the participants who had “done the month” (n = 65), when asked if they felt that they were not able to “do the month” completely because they had to go back to work, 12 (18.5%) strongly agreed or agreed, 5 (7.7%) were neutral, 35 (53.8%) disagreed or strongly disagreed, and 13 (20.0%) said this question was not applicable. Out of the participants who had “done the month” (n = 65), when asked if they were, overall, able to follow “DTM” practices, 3 (4.6%) strongly disagreed or disagreed, 13 (20.0%) were neutral, and 49 (75.4%) agreed or strongly agreed. Out of the participants who had “done the month” (n = 65), when asked if their doctor prevented them from “DTM” because they were required to come to health appointments after giving birth, 5 (7.7%) said “very much” or “quite a bit,” 4 (6.2%) said “somewhat,” 37 (56.9%) said “a little bit” or “not at all,” and 19 (29.2%) said “not applicable.”

### Attitudes Towards Doing the Month

Out of the participants who had “done the month” (n = 65), when asked if they enjoyed DTM overall, 17 (26.2%) said “not at all” or “a little bit,” 15 (23.1%) said “somewhat,” and 33 (50.8%) said “quite a bit” or “very much.” Out of the participants who had “done the month” (n = 65), when asked if they believed that “DTM” helped them to avoid sadness after giving birth, 14 (21.5%) said “not at all,” 10 (15.4%) said “a little bit,” 17 (26.2%) said “somewhat,” 13 (20.0%) said “quite a bit,” and 11 (16.9%) said “very much.” Out of the participants who had “done the month” (n = 65), when asked if they thought that “DTM” helped them recover from childbirth, 31 (47.7%) said “very much,” 18 (27.7%) said “quite a bit,” 14 (21.5%) said “somewhat,” 1 (1.54%) said “a little bit,” 1 (1.54%) said “not at all.”

### Stress

To evaluate how stressful DTM was, participants were asked about the stress that DTM placed on themselves versus their partners. Stress items used a 5-point Likert-type scale ranging from “not at all” [5] to “very much” [1]. ANOVA results indicated participants varied significantly in their stress levels by several demographic variables: place of birth (p < 0.044), year immigrated to the U.S. (p < 0.003), primary language spoken at home (p < 0.042), and self-reported spoken English level (p < 0.046). Results are described below and displayed in Table 2.

**Table 2:** Statistically significant demographic variables for stress while “Doing the Month”

Variables	Variable category	Mean ± SD	ANOVA F test p-value	Significant post hoc tests *, with effect sizes **
Place of birth	U.S.	2.80 ± 1.35	0.044	Vietnam vs. U.S. (D = 1.33)
	Other	4.07 ± 1.08		
	Vietnam	4.34 ± 1.11		
	China or Hong Kong	4.12 ± 1.01		
Immigration year	1975–2000	3.68 ± 1.34	0.003	2001–2017 vs. Not Immigrant (D = 1.61)
	2001–2017	4.32 ± 0.90		
	Not immigrant	2.80 ± 1.35		
Primary language spoken at home	English	2.80 ± 1.48	0.042	Chinese vs. English (D = 1.33)
	Other	3.90 ± 0.97		
	Vietnamese	4.20 ± 1.13		
	Chinese	4.23 ± 1.02		
Self-reported spoken English level	Not well or not at all	4.28 ± 0.88	0.046	
	Well or very well	3.72 ± 1.37		

*Tukey post hoc testing, p < 0.05; **Cohen’s D effect size

#### Stress Versus Place of Birth

For place of birth, participants born in the U.S. (Mean = 2.80, SD^1^ = 1.35) and other countries (Mean = 4.01, SD = 1.08) (i.e., other than Vietnam and China or Hong Kong) were more likely to feel that “DTM” was stressful, compared to participants born in China or Hong Kong (Mean = 4.12, SD = 1.01). Participants born in Vietnam (Mean = 4.34, SD = 1.11) were less likely to feel that DTM was stressful, compared to participants born in China or Hong Kong.

#### Stress Versus Immigration Year

Respondents who immigrated to the U.S. between 1975 and 2000 (Mean = 3.68, SD = 1.34) and respondents who were not immigrants (Mean = 2.80, SD = 1.35) were more likely than respondents who immigrated between 2001 and 2017 (Mean = 4.32, SD = 0.90) to feel that DTM was stressful.

#### Stress Versus Primary Language Spoken at Home

Survey participants who primarily spoke English at home (Mean = 2.80, SD = 1.48), Vietnamese (Mean = 4.20, SD = 1.13), or other languages (Mean = 3.90, SD = 0.10) (i.e., other than Chinese) at home were all more likely than Chinese-speakers (Mean = 4.23, SD = 1.02) to find DTM stressful.

#### Stress Versus Self-reported Spoken English Level

Respondents who self-reported their spoken English ability level as “not well” or “not at all” (Mean = 4.28, SD = 0.88) were less likely than respondents with a reported “well/very well” spoken English ability level (Mean = 3.72, SD = 1.37) to find DTM stressful.

### Provider Knowledge

Three items^2^ assessed participants’ opinions of how knowledgeable their healthcare provider was about “DTM” practices, and these items were combined to form a single measure after showing adequate internal consistency reliability (Cronbach’s alpha = 0.89). Provider Knowledge item responses ranged from “not at all” [1] to “very much” [5]. ANOVA results indicated that participants’ views of their provider’s DTM knowledge differed significantly by age (p < 0.029). Respondents who were older (≥ 45 years) felt that their healthcare provider was more knowledgeable about “DTM” practices (Mean = 3.881, SD = 1.037) than younger respondents (ages 17–44) (Mean = 2.968, SD = 1.352).

### Support While Doing the Month

Two items [(1) When you were DTM, you had family support to help you. (2) When you were DTM, you had childcare support to help you.] Assessed the level of support that participants received while “DTM,” and these items were combined to form a single measure after showing adequate internal consistency reliability (Cronbach’s alpha = 0.73). Support while DTM item responses ranged from “never” [1] to “always” [5]. ANOVA results indicated that participant support while DTM differed significantly by the primary language spoken at home (p < 0.029). Respondents who spoke English (Mean = 3.10, SD = 1.14) or other languages (i.e., other than Vietnamese or Chinese) (Mean = 3.14, SD = 1.55) were less likely to have family and childcare support while “DTM” compared to those who spoke Chinese (Mean = 4.15, SD = 1.09). Vietnamese-speakers were more likely (Mean = 4.27, SD = 0.86) to have support compared to Chinese-speakers.

### Pressure from Older Generation to Participate in DTM

Participants were asked how much they agreed with the following statement: You believe that if it were not for the older generation in your family, you would not have participated in “DTM.” ANOVA results indicated that pressure from the older generation on participants to participate in DTM differed significantly by several demographic characteristics: place of birth (p < 0.020), self-identified race/ethnicity (p < 0.022), primary language spoken at home (p < 0.003), and self-reported spoken English level (p < 0.011). Respondents who were in born in countries other than China (Vietnam Mean = 2.93, SD = 1.64; Other Countries Mean = 2.79, SD = 1.85; U.S. Mean = 1.80, SD = 0.84) were more likely to feel pressure from older generations to participate in DTM compared to those who spoke Chinese (Mean = 3.83, SD = 1.47). Those that identified as Vietnamese (Mean = 2.67, SD = 1.63) or Other Race/Ethnicity (Mean = 2.50, 1.73) were more likely to feel pressure from older generations to participate in DTM than those that identified as Chinese (Mean = 3.71, SD = 1.51). Vietnamese speakers (Mean = 2.54, SD = 1.56) and other language speakers (Mean = 1.89, SD = 1.45) were more likely to feel pressure from older generations than English speakers (Mean = 3.40, SD = 1.67) and Chinese speakers (Mean = 3.80, SD = 1.49). Those who spoke English well or very well (Mean = 2.60, SD = 1.68) were more likely to feel pressure from older generations to DTM than those who spoke English not well or not at all (Mean = 3.65, SD = 1.51). Results are shown in Table 3.

**Table 3:** Statistically significant demographic variables for older generation pressuring participant to “Do the Month”

Variables	Variable Category	Mean ± SE	ANOVA F test p-value	Significant post hoc tests*, with effect sizes**
Place of birth	U.S.	1.80 ± 0.84	0.020	China or Hong Kong vs. U.S. (D = 1.45)
	Other	2.79 ± 1.85		
	Vietnam	2.93 ± 1.64		
	China or Hong Kong	3.83 ± 1.47		
Self-identified race/ethnicity	Other	2.50 ± 1.73	0.022	
	Vietnamese	2.67 ± 1.63		
	Chinese	3.71 ± 1.51		
Primary language spoken at home	English	3.40 ± 1.67	0.003	Chinese vs. Other (D = 1.29)
	Other	1.89 ± 1.45		
	Vietnamese	2.54 ± 1.56		
	Chinese	3.80 ± 1.49		
Self-reported spoken English level	Not well or not at all	3.66 ± 1.51	0.113	
	Well or very well	2.60 ± 1.68		

*Tukey post hoc testing, p < 0.05; **Cohen’s D effect size

## Discussion

Overall, “DTM” is common in this study population and a regular part of women’s postpartum timelines, even if respondents did not necessarily know the practice by name. Even before sorting through demographic or immigrant status, the majority of respondents who had given birth had participated in “DTM” (60.7%). The majority (70.1%) of study participants also reported knowing other women who had “done the month.” This may represent a high prevalence of DTM participation beyond the survey’s respondents; that is, going out into their communities, and including women who may not utilize the FQHC in which this study was conducted. The prevalence was better captured when providing an explanatory paragraph of “DTM,” as this almost doubled the number of participants who had heard of the practice. This was made especially clear when creating the Vietnamese version of the survey as the authors found that there was no particular phrase used to describe this practice.

Demographic variables that were significantly correlated with participation in “DTM” included: place of birth, self-identified race/ethnicity, and primary language spoken at home. The fact that not all demographic variables were significant reemphasizes the importance of not assuming who is likely to participate or not participate in DTM. Nonetheless, an overwhelming majority of Chinese and Vietnamese immigrant women in this study had participated in “DTM” (Table 1). Compared to other Asian immigrants, Chinese immigrant women–defined as those born in China, who primarily speak Chinese at home, and who self-identify as ethnically Chinese–were the most likely to have participated in “DTM” postpartum practices. They were very unlikely to find “DTM” stressful, and Vietnamese immigrants felt “DTM” was even less stressful than did Chinese immigrants. The fact that Vietnamese immigrants felt they had the most social support may explain why they felt DTM was the least stressful. Primary Chinese speakers felt they had more social support than primary English speakers felt that they had during the postpartum period. Chinese immigrants were also the least likely to feel that, if it were not for the older generation, they would not have participated in “DTM.” This suggests that social support is important and impacts a woman’s experience during this postpartum ritual.

Although sometimes viewed as a restrictive and isolating practice, “DTM” appears to be well-received among women who participate. Participants generally thought that “DTM” was stressful, more so for themselves than for their partners. Women’s attitudes towards whether they enjoyed the practice were very mixed and ranged across all the available response options. However, most respondents did report enjoying “DTM.” In order to properly address our population’s immigrant experiences, it was important for this study to make a distinction between how much women enjoy this practice and how culturally important this practice is for some women. Immigrants living in the United States may experience conflicting values between acculturation and enculturation [28]. Western cultures place a greater emphasis on individual beliefs and personal values, thus conflicting with Eastern, more collectivist cultures which emphasize parental authority and conformity to familial/cultural traditions and norms [20, 21]. Personal values and familial/cultural traditions do not necessarily align, so understanding the importance of the traditional practices like “DTM” may not be enough to ensure adherence to it [22]. Distinguishing women’s participation as a result of their personal preferences and what they consider to be favorable or acceptable, as opposed to the result of what their culture or family forces them to do, sheds more light on their attitudes towards “DTM.”

### Clinical Implications

Providers should be aware of and familiar with common components that may affect postpartum health outcomes for mothers and infants. This includes but is not limited to the avoidance of showering and brushing teeth, drinking rice wine, and maintaining bedrest while not leaving the home. As patients share the DTM practices they may participate in, providers should provide education and counseling on potential negative health outcomes.

Common DTM practices around hygiene include avoiding brushing one’s teeth and showering. The American Dental Association recommends brushing one’s teeth twice a day to prevent caries and gingivitis, and evidence shows that periodontal disease is associated with heart disease [23, 24]. Being unable to shower can contribute to postpartum infections, and being unable to leave the house may contribute to postpartum depression in the mother, especially for women who delivered by Cesarean sections [25].

Another common DTM practice is drinking rice wine. Depending on the amount of time that has passed between rice wine consumption and nursing, drinking rice wine or consuming foods cooked with rice wine could affect developing infants, as well as increase time needed for breastfeeding patients to lactate [26, 27].

Additionally, “DTM” did not seem to prevent most women from seeing their doctor in the month after giving birth. However, there was a substantial number of women (one-fourth) who felt that “DTM” did prevent them from seeing their doctor during the thirty days postpartum. These women and their newborns may be the most vulnerable in terms of postpartum depression and other negative health outcomes [25]. A month of bedrest could increase an already elevated risk for deep vein thrombosis [28]. Remaining indoors may also prevent adequate sun exposure and thus a vitamin D deficiency, which can further contribute to risk of depression [29]. Vitamin D supplements during this time may help to address this concern. Additionally, providers should offer telemedicine or home-visit options for women who cannot leave their homes during the month after giving birth to ensure timely care is available during in dynamic period following childbirth. In the spring of 2020, telemedicine appointments significantly increased due to the coronavirus disease 2019 pandemic [30]. Telemedicine is expected to continue advancing even beyond the pandemic, which may help to ameliorate cultural conflicts of “DTM” [31].

When relevant, providers should ask patients whether they participate in DTM practices by providing a list of concrete examples of common components (e.g., not showering, not eating cold foods, not leaving the home) for patients to review rather than using the general English translation of “DTM.” Providers should also ask patients how long they plan on participating in each component. To avoid limiting patients’ responses, providers should hold space for patients to share any postpartum practices that were not already mentioned.

A discussion on any barriers the patient may experience that prevent the patient from participating in certain components of DTM will inform the provider of other needs to address in working towards a satisfying and successful postpartum recovery period. Providers working with Asian communities in which the practice could affect postpartum health may need to screen all patients, regardless of immigration status or self-identified race/ethnicity. This conversation should happen in early prenatal appointments so that the patient, any individuals who may support the patient during the postpartum recovery period, and the provider may explore options and prepare for them prior to delivery.

The findings of this study are not limited to Asian immigrants. There are many other similar variations on this postpartum practice to be found in other cultures. For example, many Latin American women participate in the “cuarenteña,” during which some women drink a beer each morning, believing it aids in breastmilk production. Some Indian women participate in “magu-bananthi aarike” and may be restricted to only ingesting bread, coffee, and milk during the first two days after giving birth. This example illustrates how beverage consumption alone as a component of postpartum practices may be very nuanced. In order to provide the best care, obstetricians, midwives, doulas, nurses, pediatricians, internal medicine, family medicine doctors, and all other medical providers who come in contact with individuals who are pregnant or recently pregnant should all be aware of these various practices, be trained to ask what components they are participating in when relevant, and be knowledgeable about how to provide appropriate education and medical care for patients who are or will be participating in their respective postpartum practices. Medical and residency training should include curricula regarding traditional postpartum rituals, including education on common components, recommendations that work with these components, and the cultural significance of these rituals.

### Recommendations for Future Studies

A large majority of women also felt like “DTM” helped them to recover from childbirth; only 1 (1.54%) person said that it did not help at all. Further qualitative studies are required to elucidate this finding. In addition, further retrospective electronic health record analyses are also required to determine whether women who “did the month” actually had a better or worse recovery outcome compared to those that did not.

According to Pew Research Center estimates, Asian immigrants are projected to become the largest foreign-born group in the U.S. by 2055, surpassing Hispanics [32]. “DTM” is a very common practice among Chinese and Vietnamese immigrants that health providers should be aware of in order to provide and promote culturally-responsive postnatal health of both the infant and mother. Conversely, the strong aspects of social support and emphasis on recovery may be protective of postpartum depression. Future retrospective studies evaluating the correlation between DTM and postpartum health outcomes are sorely needed.

Limitations of this study include small sample size, convenience and cross-sectional sampling, recruiters who were unable to speak Cantonese, and many Mongolian and Burmese patients who were interested in participating were unfortunately unable to take the survey due to the lack of appropriate language translations. Statistical analyses were also limited by the small sample size. Analyses were therefore exploratory in nature and should be confirmed with a larger sample.

Strengths of this study include: the first study related to “DTM” practices in the U.S., the second study ever to study immigrants’ participation in “DTM,” and the availability of survey instruments in both Chinese and Vietnamese.

## Conclusions

The majority of immigrant Asian women at this FQHC have heard of and participated in “DTM.” While DTM was stressful for some women, many enjoyed participating in this practice and many more found it helpful for recovery from childbirth. Asian immigrants are projected to become the largest foreign-born group in the U.S. by 2055. DTM is a common practice that health providers should be aware of in order to promote postnatal health of both infant and mother. Drinking rice wine, recommended in DTM practices, could affect developing infants and may need to be addressed in breastfeeding patients. Being unable to shower or leave the house for a month may contribute to postpartum infections or depression in the mother.

## Footnotes

1 SD = standard deviation.

2 Your healthcare provider was well informed about “doing the month” practices. Your healthcare provider was able to answer your questions about DTM. Overall, your healthcare provider’s knowledge about DTM was ___.
